# Etiology and characteristics of patients with bronchiectasis in Taiwan: a cohort study from 2002 to 2016

**DOI:** 10.1186/s12890-020-1080-7

**Published:** 2020-02-18

**Authors:** Hung-Yu Huang, Fu-Tsai Chung, Chun-Yu Lo, Horng-Chyuan Lin, Yu-Tung Huang, Chih-Hsin Yeh, Chang-Wei Lin, Yu-Chen Huang, Chun-Hua Wang

**Affiliations:** 1 Division of Pulmonary and Critical Care, Department of Internal Medicine, Saint Paul’s Hospital, Taoyuan, Taiwan; 2 Department of Thoracic Medicine, Chang Gung Memorial Hospital, and College of Medicine, Chang Gung University, 199 Tun-Hwa North Road, Taipei, Taiwan; 3 College of Medicine, Chang Gung University, Taoyuan, Taiwan; 4 Center for Big Data Analytics and Statistics, Chang Gung Memorial Hospital, Taoyuan, Taiwan; 5 Graduate Institute of Clinical Medical Sciences, College of Medicine, Chang Gung University, Taoyuan, Taiwan

**Keywords:** Comorbidity, Etiology, Microbiology, Non-cystic fibrosis bronchiectasis

## Abstract

**Background:**

Bronchiectasis is a chronic infectious respiratory disease with diverse causes and ethnic or geographic differences. However, few large-scale studies of its etiology have been conducted in Asia. This study aimed to determine the etiology and clinical features of bronchiectasis in Taiwan.

**Methods:**

This longitudinal cohort study investigated the etiology and clinical features of newly diagnosed non-cystic fibrosis bronchiectasis patients from January 2002 to December 2016. The clinical, functional and microbiological data of patients were retrieved from the Chang Gung Research Database, which includes seven medical facilities throughout Taiwan. The index date was the date of the first bronchiectasis diagnosis. Known diseases that were diagnosed before the index date were regarded as etiologies of bronchiectasis.

**Results:**

The cohort comprised 15,729 adult patients with bronchiectasis. Idiopathic (32%) was the most common cause, followed by post-pneumonia (24%). Other causes included post-tuberculosis (12%), chronic obstructive pulmonary disease (14%), asthma (10%), gastroesophageal reflux disease (2%) and rheumatic diseases (2%). At diagnosis, 8487 patients had sputum culture. *Pseudomonas aeruginosa* (5.3%) was the most common bacteria, followed by non-tuberculosis mycobacteria (3.6%), *Haemophilus influenzae* (3.4%) and *Klebsiella pneumoniae* (3.1%), but 6155 (72.1%) had negative sputum cultures. Patients with post-tuberculosis had a higher sputum isolation rate of non-tuberculosis mycobacteria than *P. aeruginosa*. Patients with post-tuberculosis and post-pneumonia bronchiectasis had a higher frequency of chronic lung infection than other groups (*p* < 0.05). Clinical characteristics, such as gender, lung function, comorbidities and microbiology, were significantly different between idiopathic and known etiologies.

**Conclusions:**

Idiopathic, post-infection and tuberculosis constitute major bronchiectasis etiologies in Taiwan. Clinical characteristics and sputum microbiology were distinct among separate etiology phenotypes.

## Background

Non-cystic fibrosis (CF) bronchiectasis is a chronic pulmonary disease characterized by repeated infection and subsequent destruction to the airway wall, thus resulting in an irreversible dilatation of a portion of the bronchial trees. Bronchiectasis can occur from early childhood to late adulthood, but the average age in US, European, and Australasian cohorts is between 60 and 70 years, and the incidence of bronchiectasis is positively associated with age [1–3]. Geographic variation exists in the etiology, incidence, prevalence and clinical features [4–9]. The most common cause of bronchiectasis is post-infection and other causes include chronic obstructive pulmonary disease (COPD), asthma, connective tissue disease and immunodeficiency [4–6]. Previous studies have reported a lower incidence of COPD and asthma as etiologies of bronchiectasis in Asia than in Western countries [10, 11]. However, tuberculosis is the main cause of post-infectious bronchiectasis in Asian populations [10, 11]. Most types of bronchiectasis are also dominant in Asian regions and reports of their characteristics vary widely because of selection or referral biases, and the extent of testing to seek a diagnosis of bronchiectasis is underreported [7]. A population-based study is therefore needed to evaluate the etiological spectrum of bronchiectasis in Asia.

The microbiology of bronchiectasis also varies by geography [7]. *Pseudomonas aeruginosa* and *Haemophilus influenzae* have been identified as the most common bacteria and their presence was associated with frequent exacerbations of bronchiectasis [12]. Nontuberculous mycobacteria (NTM) represent an increasingly common pathogen of bronchiectasis and the coinfection of NTM and *P. aeruginosa* is associated with more exacerbations and lung function decline [13]. The distribution of pathogens detected in bronchiectasis varies among populations [7]. However, the distribution of pathogens associated with various etiologies has not been investigated deeply due to the limited patient numbers in previous studies.

Nationwide population studies have been conducted to estimate the incidence, prevalence and health-care burden of bronchiectasis [1, 2]. In Asia, there are only limited series data regarding the etiology of bronchiectasis [14]. Thus, this study evaluated the etiology spectrum, microbiology and comorbidity of bronchiectasis in a multicenter bronchiectasis cohort in Taiwan over a 15 year period.

## Method

### Data source

In this study, we used the Chang Gung Research Database (CGRD) to construct a multicenter bronchiectasis cohort from 2002~2017. The CGRD is an electronic medical record database of Chang Gung Memorial Hospital (CGMH), which is the largest hospital system in Taiwan comprising three medical centers (Linkuo branch, Taipei branch, and Kaohsiung branch) and four regional hospitals (Taoyuan branch, Keelung branch, Keelung branch and Yunlin branch). These hospitals are located across Taiwan (http://www.chang-gung.com/en/index.aspx). The data in the CGRD include demographic data, inpatient and outpatient data, diagnostic codes, details of prescriptions and reports of microbiological, image and functional examinations [15]. More detailed information about CGRD had been reported in a previous article [16].

### Bronchiectasis cohort

Initially, patients with diagnoses of bronchiectasis between January 2002 and December 2017 were included in the bronchiectasis group. The diagnosis code of bronchiectasis was according to the International Classification of Diseases, Ninth Clinical Modification (ICD-9-CM) 494.0 or 494.1, or 10th Revision (ICD-10) J47. The diagnosis of bronchiectasis was confirmed by the interpretation of chest radiography or high resolution computed tomography (HRCT), which was independently reviewed by a radiologist. The chest radiographs from the time of diagnosis of bronchiectasis using the criteria included the presence of increased pulmonary markings, honeycomb-like structures, atelectasis, and pleural changes [17]. The presence of bronchiectasis of HRCT was confirmed with the finding of abnormally dilated bronchi near the peripheral lung fields, failure of bronchi to taper with increasing distance from the hilum, or cystic dilatations of the bronchi with or without fluid levels [18]. 10,724 subjects had HRCT images, while 5005 patients were confirmed as bronchiectasis by chest radiographs. The average portion of patients having HRCT images increased annually (2002–2005: 43.1%; 2006–2010: 71.0%; 2011–2016: 90.3%). Symptoms of bronchiectasis in the presence of chronic cough with the production of purulent sputum, hemoptysis, recurrent fever, and pleurisy were recorded and identified by the CGRD database. The diagnosis included both clinical symptoms and radiological images of bronchiectasis. Patients with at least two bronchiectasis diagnoses on different dates in an outpatient visit record or any one from hospitalization were included in the cohort. The index date was defined as the diagnosis date of bronchiectasis. The cohort only included patients aged ≥18 years. Demographic data were collected. We retrieved the image reports, then identified and calculated the total number of affected lobes. The disease extent of 2 was set as a cut-off according to previously described method [19]; thus, the affected bronchiectasis site (< 2 lobes affected, 2 lobes, or ≥ 3 lobes affected) was determined. The lingual lobe was considered a separate lobe.

### Etiology, comorbidity, and microbiology

In total, our cohort included 15,729 cases. For bronchiectasis patients, clinicians mostly reviewed history of pulmonary tuberculosis and pneumonia, and performed infection workups (sputum culture and mycobacterial culture) to define a postinfective etiology of bronchiectasis. As the next step, in the absence of a positive history of previous severe respiratory infection, the clinician surveyed an association between bronchiectasis and other diseases, including COPD, asthma, gastroesophageal reflux (GERD), and connective tissue disease. Bronchiectasis associated with COPD was diagnosed by a smoking history of 10 pack-years and airflow obstruction (FEV_1_/FVC ratio < 0.7) according to the American Thoracic Society (ATS) guidelines [20]. Bronchiectasis associated with asthma was diagnosed in patients without postinfective bronchiectasis and who had severe asthma according to the Global Initiative for Asthma guidelines [21]. The diagnosis of GERD was retrieved by the ICD code, which was made by clinicians and based on symptoms and gastroscopy. In cases of clinical suspicion of immunodeficiency and connective tissue diseases, serum IgG, IgA, IgM, total IgE, specific IgE, and autoimmunity testing, including antinuclear antibodies, extractable nuclear antigens, antineutrophil cytoplasmic antibodies, rheumatoid factor, and anticitrullinated protein antibody, were measured. Thus, only 1505 patients completed the immunoglobulin tests. When primary ciliary dyskinesia was suspected, such as recurrent sinusitis and/or chronic otitis media, nasal mucociliary clearance was measured by the saccharin test. α1-Antitrypsin was evaluated when HRCT revealed the presence of emphysema affecting the lower lobes. Sweat tests were requested if signs and symptoms suggestive of cystic fibrosis were present. Bronchiectasis-associated etiologies were defined as including a history of pneumonia (bacteria, fungus, pertussis and measles) with at least 7 days of antibiotic prescriptions, pulmonary tuberculosis, immune deficiency, asthma, connective tissue disorders, gastroesophageal reflux (GERD), and COPD. Bronchiectasis was defined as idiopathic if the cause was unidentifiable despite a full etiological study before the index date.

Comorbidities obtained from CGRD diagnoses (ICD-9-CM and ICD-10) within 12 months before or after definite diagnosis date of bronchiectasis included ischemic heart disease, cerebrovascular disease, diabetes, liver disease, chronic renal failure, solid tumor and hematological malignancy [22, 23]. We collected sputum microbiology reports within 6 months before or after the index date. The sputum could be taken either in stable state or during exacerbation. Sputum cultures for both bacteria and mycobacteria were performed for each patient with productive cough. Identification of microorganisms and susceptibility testing were taken. Chronic infection was defined as isolation of the same pathogen in two or more cultures, at least 3 months apart within a 12-month span [24]. Pulmonary function test including forced expiratory volume in 1 sec (FEV_1_), forced vital capacity (FVC), and FEV_1_/FVC ratio, was performed with a spirometer according to the ATS and the European Respiratory Society (ERS) criteria [25].

## Statistics

We used the analysis of variance (ANOVA) test (for more than 3 groups) and Student’s *t* test to compare numerical data. The chi-square test was used to compare independent categorical data and a chi- square test for trend model was used to evaluate the proportional change between three periods (2002–2006, 2007–2011, and 2012–2016). Data processing and analyses were performed using SAS Enterprise Guide version 7.1 (SAS Institute, Inc.). For all analyses, *p* value < .05 was considered statistically significant.

## Results

**Fig. 1:**
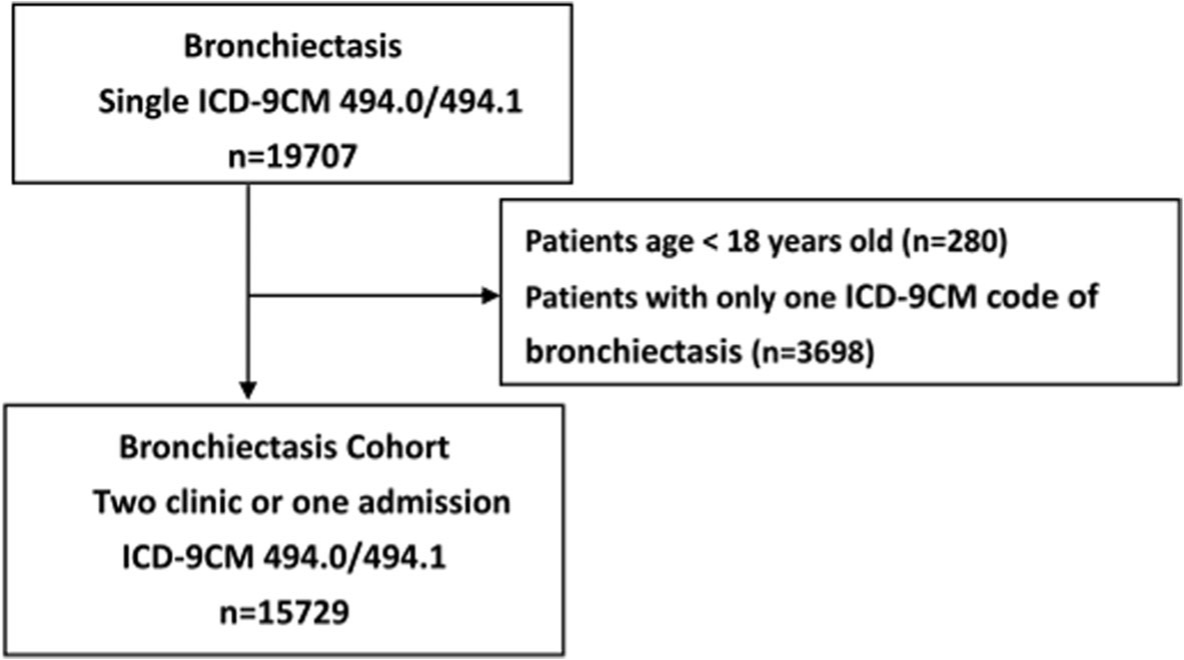
Bronchiectasis cohort flow diagram, 2002 to 2016. ICD-9-CM: International Classification of Diseases, Ninth Revision, Clinical Modification

In the CGRD, we received data on 19,707 patients with at least one ICD-9-CM 494.0 or 494.1 claim from 2002 to 2016. We excluded patients with only one ICD-9-CM 494.0 or 494.1 claim (*n* = 3698) and those aged < 18 (*n* = 280). A total of 15,729 patients who had at least two ICD claims in outpatient visits or any one ICD claim from hospitalization was included in our bronchiectasis cohort (Fig. 1). After the diagnosis of bronchiectasis, most of the patients had follow-up in our clinic. Only 9.2% had only once clinic follow-up. The mean time of follow-up was 3.8 years. A total of 85% of patients were followed up regularly and 66% of the cohort had at least 3 follow-up visits at our clinic in 1 year.

The etiologies of newly diagnosed bronchiectasis patients are listed in Table 1. Post-infection (36.3%) was the most common cause (pneumonia (23.9%) and tuberculosis (12.4%)). Other causes included COPD (14.5%), asthma (10.6%), GERD (2.5%), connective tissue diseases (2.3%) and immunodeficiency (1.3%). Idiopathic bronchiectasis was observed in 32.0% of the cohort. The connective tissue disease group consisted of rheumatoid arthritis (*n* = 282, 77%), *systemic lupus erythematosus* (*n* = 62, 17%), sclerosis (*n* = 8, 2%), dermatomyositis (*n* = 9, 2%), and polymyositis (*n* = 6, 2%). Among the GERD group, 148 (37.1%) performed gastroscopy and 341 (85.5%) received medication for GERD within 1 year of diagnosis.

**Table 1:** Etiology of non-CF bronchiectasis

Total 2002~2016	*N* = 15,729
Etiology	
Idiopathic	5036 (32.0)
Post-infection	
Tuberculosis	1950 (12.4)
Pneumonia	3766 (23.9)
Immunodeficiencies	206 (1.3)
COPD	2287 (14.5)
Asthma	1664 (10.6)
Connective tissue disease	367 (2.3)
GERD	399 (2.5)
Other^a^	54 (0.3)

Note: Data are presented as number (percentage)

*COPD* Chronic obstructive pulmonary disease, *GERD* Gastroesophageal reflux disease

^a^Other include inflammatory bowel disease, Yellow nail syndrome, Alpha-1 antitrypsin deficiency, tracheo-oesophageal fistula, primary ciliary dyskinesia

### Clinical characteristics

**Fig. 2:**
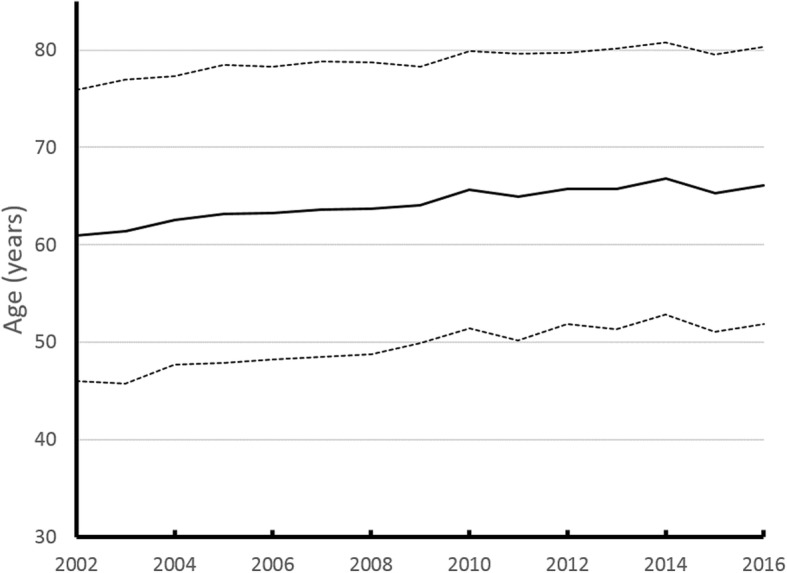
Age trend of bronchiectasis in Taiwan since 2002~2016

Among the CGRD bronchiectasis cohort, the average age at diagnosis was 64 years and 49.5% were 65 years or older. In the gender distribution, 54.9% were women. The mean duration for clinical follow-up was 3.8 years. The first diagnosis was made during hospitalization in 26.9% of patients and all others were diagnosed in outpatient clinics. The mean age of newly diagnosed patients increased significantly with time during the 15-year period (Fig. 2).

The clinical characteristics of separate groups of bronchiectasis patients are summarized in Table 2. Patients with COPD were the oldest and had the lowest FEV_1_ at the initial diagnosis. Patients with post-tuberculosis and other infection had higher rates of first diagnosis during hospitalizations and had the most comorbid malignancy. Patients with immunodeficiency were predominantly female (73.8%) and had more affected lobes and less comorbid malignancy. Idiopathic bronchiectasis was the youngest at diagnosis and had higher FEV_1_ and the shortest follow-up duration.

**Table 2:** Clinical characteristics by etiology of bronchiectasis

	Total (*N* = 15,729)	Idiopathic (*n* = 5036)	Tuberculosis (*n* = 1950)	Other infection (*n* = 3766)	Immuno-deficiencies (*n* = 206)	COPD (*n* = 2287)	Asthma (*n* = 1664)	CTD (*n* = 367)	GERD (*n* = 399)	*p* value
Age at diagnosis (mean)	64.0 ± 14.8	60.0 ± 15.5	64.5 ± 14.9	65.5 ± 14.9	64.7 ± 13.2	67.6 ± 12.6	60.2 ± 14.7	64.7 ± 13.4	60.6 ± 14.0	< 0.001
Age at diagnosis										< 0.001
< 40 yr	1041(6.6)	459(9.1)	127(6.5)	204(5.4)	10(4.9)	55(2.4)	148(8.9)	17(4.6)	16(4.0)	
40–65 yr	6908(43.9)	2498(49.6)	743(38.1)	1440(38.2)	124(60.2)	839(36.7)	823(49.5)	184(50.1)	239(59.9)	
> 65 yr	7780(49.5)	2079(41.3)	1080(55.4)	2122(56.3)	72(35.0)	1393(60.9)	693(41.6)	166(45.2)	144(36.1)	
Gender, female	8631(54.9)	3005(59.7)	933(47.8)	1961(52.1)	152(73.8)	924(40.4)	1106(66.5)	282(76.8)	244(61.2)	< 0.001
HRCT Bronchiectasis site										
< 2 lobes	2317(56.2)	685(59.8)	311(52.9)	628(55.1)	24(39.3)	305(56.3)	239(56.8)	50(57.5)	71(55.4)	< 0.001
2 lobes	1026(24.9)	259(22.6)	156(26.5)	296(25.9)	24(39.3)	128(23.7)	103(24.4)	22(25.3)	34(26.6)	< 0.001
> 2 lobes	777(18.9)	201(17.6)	121(20.6)	217(19.0)	13(21.4)	108(20.0)	79(18.8)	15(17.2)	23(18.0)	< 0.001
Pulmonary function										
Restriction	2301(28.5)	658(32.0)	275(30.4)	718(36.2)	47(39.5)	0(0%)	481(44.3)	63(28.5)	51(25.2)	< 0.001
Normal or Obstruction										
FEV_1_ > 80	3286(40.7)	1055(51.3)	308(34.0)	667(33.6)	57(47.9)	572(39.2)	381(35.1)	99(44.8)	136(67.3)	< 0.001
FEV_1_ 50–80	1539(19.1)	259(12.6)	193(21.3)	353(17.8)	10(8.4)	496(34.0)	158(14.5)	48(21.7)	11(5.4)	< 0.001
FEV_1_ < 50	938(11.6)	84(4.1)	130(14.3)	246(12.4)	5(4.2)	390(26.8)	67(6.2)	11(5.0)	4(2.0)	< 0.001
Microbiology at diagnosis										
Negative	6289(75.1)	1763(78.9)	1001(71.2)	1766(71.9)	88(84.6)	816(77.1)	539(75.8)	134(74.4)	159(81.5)	< 0.001
*Pseudomonas aeruginosa*	441(5.3)	96(4.3)	54(3.8)	166(6.8)	4(3.8)	63(6.0)	42(5.9)	7(3.9)	8(4.1)	0.002
NTM	304(3.6)	89(4.0)	101(7.2)	47(1.9)	3(2.9)	23(2.2)	28(3.9)	5(2.8)	8(4.1)	< 0.001
*Haemophilus influenzae*	282(3.4)	61(2.7)	27(1.9)	107(4.4)	3(2.9)	35(3.3)	34(4.8)	9(5.0)	6(3.1)	0.001
*Klebsiella pneumoniae*	259(3.1)	68(3.0)	27(1.9)	100(4.1)	0(0.0)	34(3.2)	21(3.0)	3(1.7)	4(2.1)	0.007
*Haemophilus parainfluenzae*	113(1.3)	34(1.5)	12(0.9)	35(1.4)	1(1.0)	8(0.8)	16(2.3)	5(2.8)	2(1.0)	0.089
Fungus	102(1.2)	26(1.2)	22(1.6)	32(1.3)	0(0.0)	11(1.0)	6(0.8)	3(1.7)	2(1.0)	0.787
Tuberculosis	86(1.0)	3(0.1)	80(5.7)	3(0.1)	0(0.0)	0(0.0)	0(0.0)	0(0.0)	0(0.0)	< 0.001
*Staphylococcus aureus*	73(0.9)	13(0.6)	11(0.8)	34(1.4)	1(1.0)	7(0.7)	3(0.4)	3(1.7)	1(0.5)	0.086
Chronic infection^a^	195(10.7)	25(12.8)	48(24.6)	68(34.9)	2(1.0)	27(13.9)	16(8.2)	4(2.1)	5(2.6)	< 0.001
First diagnosis										< 0.001
Clinic	11,498(73.1)	4079(81.0)	1180(60.5)	2367(62.9)	157(76.2)	1644(71.9)	1407(84.6)	284(77.4)	346(86.7)	
Hospitalization	4231(26.9)	957(19.0)	770(39.5)	1399(37.2)	49(23.8)	643(28.1)	257(15.5)	83(22.6)	53(13.3)	
BACI score	4.75 ± 5.24	2.62 ± 4.37	5.8 ± 5.86	5.78 ± 5.65	5.74 ± 5.48	6.88 ± 4.9	4.64 ± 4.12	6.29 ± 5.03	2.91 ± 4.27	<.0001
Comorbidity										
Ischemic heart disease	677(4.3)	168(3.3)	93(4.8)	187(5.0)	6(2.9)	127(5.6)	62(3.7)	13(3.5)	18(4.5)	< 0.001
Cerebrovascular disease	638(4.1)	167(3.3)	92(4.7)	213(5.7)	3(1.5)	98(4.3)	35(2.1)	12(3.3)	13(3.3)	< 0.001
Diabetes	1066(6.8)	258(5.1)	142(7.3)	350(9.3)	9(4.4)	182(8.0)	88(5.3)	16(4.4)	17(4.3)	< 0.001
Liver disease	630(4.0)	185(3.7)	96(4.9)	145(3.9)	16(7.8)	105(4.6)	54(3.2)	17(4.6)	9(2.3)	0.003
Chronic kidney disease	434(2.8)	93(1.8)	76(3.9)	123(3.3)	5(2.4)	77(3.4)	40(2.4)	12(3.3)	6(1.5)	< 0.001
Solid tumor	1115(7.1)	313(6.2)	183(9.4)	356(9.5)	8(3.9)	147(6.4)	67(4.0)	18(4.9)	15(3.8)	< 0.001
Hematological malignancy	660(4.2)	201(4.0)	86(4.4)	207(5.5)	9(4.4)	85(3.7)	46(2.8)	14(3.8)	8(2.0)	< 0.001
Follow up period, years	3.8 ± 4.2	2.3 ± 3.6	4.4 ± 4.2	4.2 ± 4.2	4.4 ± 4.4	4.4 ± 4.4	4.4 ± 4.4	5.3 ± 4.5	4.6 ± 4.1	< 0.001

Note: Data are presented as number (percentage). *CTD* Connective tissue disease, *GERD* Gastroesophageal reflux disease

Analysis of variance (ANOVA) test is used for multiple comparison

^a^Chronic infection: isolation of the same pathogen in two or more cultures, at least 3 months apart in a 12-month span

### Time trends of etiology

The etiologies of bronchiectasis exhibited a significant trend of change during the three time periods (Table 3). Post-tuberculosis bronchiectasis gradually decreased, whereas post-pneumonia bronchiectasis gradually increased from 2002 to 2006 to 2012–2016. The percentage of COPD and asthma groups showed a significant reduction from year 2002–2006 to year 2012–2016. However, the time trends of the idiopathic and immunodeficiency groups did not change significantly.

**Table 3:** The time trend in etiology of non-CF bronchiectasis

	*N* = 15,729	2002~2006 *N* = 6263	2007~2011 *N* = 4569	2012~2016 *N* = 4897	*p* value
Etiology					
Idiopathic	5036 (32.0)	1990(31.8)	1533(33.6)	1513(30.9)	0.4752
Post-infection					
Tuberculosis	1950 (12.4)	845(13.5)	575(12.6)	530(10.8)	<.0001
Pneumonia	3766(23.9)	1376(22.0)	1102(24.1)	1288(26.3)	<.0001
Immunodeficiencies	206(1.3)	75(1.2)	64(1.4)	67(1.4)	0.4077
COPD	2287(14.5)	1038(16.6)	584(12.8)	665(13.6)	<.0001
Asthma	1664(10.6)	727(11.6)	470(10.3)	467(9.5)	0.0004
Connective tissue disease	367(2.3)	128(2.0)	103(2.3)	136(2.8)	0.0119
GERD	399(2.5)	67(1.1)	122(2.7)	210(4.3)	<.0001
Other^a^	54(0.3)	17(0.3)	16(0.4)	21(0.4)	0.1574

Note: Data are presented as number (percentage),

*COPD* Chronic obstructive pulmonary disease, *GERD* Gastroesophageal reflux disease

^a^Other include inflammatory bowel disease, Yellow nail syndrome, Alpha-1 antitrypsin deficiency, tracheo-oesophageal fistula, primary ciliary dyskinesia

The statistical analysis is the chi-square (*χ*^2^) test for trend model

### Microbiology

At diagnosis, 8385 patients had sputum cultures. *P. aeruginosa* (5.3%) was the most common bacteria, followed by *non-tuberculosis mycobacteria* (NTM, 3.6%), *H. influenzae* (3.4%) and *Klebsiella pneumoniae* (3.1%). A total of 6289 (75.1%) patients had negative sputum culture at diagnosis. However, post-tuberculosis bronchiectasis had higher sputum isolation rate of NTM than *P. aeruginosa*. In total, 1824 patients received two or more cultures, at least 3 months apart in a 12-month span and 10.7% had chronic infection. The microorganisms of chronic infection are listed in Table 4. The different etiologies showed diverse microbiological patterns. Patients with post-tuberculosis and post-pneumonia bronchiectasis had a higher frequency of chronic lung infection (*p* < 0.05). Among post-pneumonia bronchiectasis, the ten most common pathogens of sputum culture during pneumonia were *P. aeruginosa* (22.4%), *K. pneumoniae* (11.5%), *H. influenza* (10.5%), *Staphylococcus aureus* (6.6%), *Haemophilus parainfluenzae* (4.4%), *Acinetobacter baumannii* (4.1%), *Streptococcus pneumoniae* (3.6%), *Acinetobacter baumannii-MDR strain* (2.5%), *Escherichia coli* (2.5%), and mold (2.2%).

**Table 4:** Microbiology of chronic infection

Repeated Sputum culture	*n* = 2255
Negative	1685 (75.0%)
*Pseudomonas aeruginosa*	143 (6.3%)
NTM	107 (4.7%)
*Klebsiella pneumoniae*	61 (2.7%)
*Staphylococcus aureus*	44 (2.0%)
*Haemophilus influenzae*	29 (1.3%)
*Acinetobacter baumannii*	22 (1.0%)
Fungus	18 (0.8%)
*Haemophilus parainfluenzae*	14 (0.6%)
*Escherichia coli*	14 (0.6%)
*Streptococcus pneumoniae*	10 (0.4%)
*Acinetobacter baumannii-*MDR strain/PDR strain	11 (0.5%)
Tuberculosis	10 (0.4%)
*K pneumoniae*-ESBL strain	8 (0.4%)
Other	79 (3.5%)

## Discussion

Our study investigated the clinical characteristics of non-CF bronchiectasis over a 15-year period in Taiwan. Bronchiectasis with different etiologies exhibited distinctive features of age, gender, microbiology and comorbidities. Our study demonstrated that postinfectious bronchiectasis was the main etiology, and it had the highest rate of chronic infection. NTM infection ranked first in post-tuberculosis bronchiectasis. With regard to the trend of newly- diagnosed bronchiectasis in Taiwan, we observed that post-tuberculosis bronchiectasis significantly decreased, whereas the cause of other infectious bronchiectasis increased during the 15-year period. Bronchiectasis caused by GERD also increased during the study period. The prevalence of bronchiectasis was also higher in the elderly and dominant in women. Taken together, the results of our study provide crucial information on the epidemiology of bronchiectasis in Asia.

The prevalence of bronchiectasis increased with patient age in previous studies [1, 2]. Likewise, we observed that 50% of patients in the bronchiectasis cohort were over 65 years old [26]. Increased age is considered a risk factor for bronchiectasis [27]. Older adults were more likely to be symptomatic and thus experience a worse quality of life. Multimorbidity is common among patients with bronchiectasis and is associated with an increased risk of hospitalization with a severe exacerbation and mortality [28], thus leading to a significant economic burden in society [29]. Our results revealed that the mean age of newly diagnosed bronchiectasis patients has risen in the past 15 years. Taiwan is an aging society and population aging is associated with increased multiple chronic conditions among people aged 65 years or older [30, 31]. Therefore, surveying the epidemiology of bronchiectasis and evaluating disease phenotypes are crucial in Taiwan to improve diagnosis and ensure adequate treatment of bronchiectasis patients, thus, reducing the negative effects on quality of life and health-care burden.

We report a 36% rate of postinfectious bronchiectasis, which is compatible with previous studies (20–70%) [6, 9–11]. A history of tuberculosis is positively associated with the presence of chronic respiratory diseases, particularly bronchiectasis [32]. The strongest associations between tuberculosis and bronchiectasis have been observed in countries with a high incidence of tuberculosis [32]. The high proportion of post-tuberculosis bronchiectasis in our cohort may be that tuberculosis is still prevalent in Taiwan. Therefore, screening for tuberculosis or tracing history of tuberculosis should be performed in newly diagnosed bronchiectasis patients. Due to the public health policy for tuberculosis control and directly observed treatment (short course), the prevalence of tuberculosis in Taiwan continued to decline from 2005 to 2017 (see https://daily.cdc.gov.tw/stoptb/CareMagChart.aspx (the incidence rate is per 100,000 people in the graph, Taiwan Centers for Disease Control website) [33]. This study also found that the incidence of post-tuberculosis declined despite that of infectious etiology overall remaining constant because the number of patients with infections other than tuberculosis increased.

Bronchiectasis is increasingly recognized as a complication of asthma and COPD, particularly those of advanced stages [34, 35]. In our cohort, COPD-associated bronchiectasis was the third most common etiologic group (15%), with the oldest age and the most severe airflow obstruction. Although a causal association has not been determined, bronchiectasis in patients with COPD has been reported to have the existence of chronic bronchial infection associated with a greater bacterial load [36, 37], an increase in both local and systemic inflammation [37, 38], and a greater frequency and severity of exacerbations [37, 39]. Thus, it may be biologically plausible that COPD, particularly infection and exacerbation, could be the cause of bronchiectasis without any other known etiology. A higher mortality was reported in bronchiectasis overlapping COPD than COPD alone [38]. Future research should investigate specific biomarkers linking COPD and bronchiectasis, such as neutrophilic inflammation, COPD phenotypes, bronchial wall thickening of image surveys, or increased susceptibility to chronic bronchial infection. Thus, we could identify the risk factors of COPD-associated bronchiectasis to determine the optimal treatment and to decrease the health-care burden. In our study, the proportion of COPD-associated bronchiectasis was higher than those previously reported in Asian populations [9–11]. A higher prevalence (20 to 52%) of COPD has been observed in patients with bronchiectasis in the United Kingdom and United States [1, 2]. Geographic variations of COPD-associated bronchiectasis still exist and future studies are needed to survey the prevalence and prognosis of this specific phenotype in Asian populations.

We reported a 32% rate of idiopathic bronchiectasis, which is comparable to previous studies (6 to 77%) [6, 9–11]. Idiopathic bronchiectasis was reported to be highly variable because of differences in enrolled study populations, criteria of etiologies and whether diagnoses are based on diagnostic exams or ICD codes. This study was based on databases of medical centers; thus, idiopathic bronchiectasis may be less common in our database compared to local medical clinics.

GERD, a possible etiology of bronchiectasis, has not been well investigated. GERD is a risk factor for recurrent microaspiration, which may cause airway irritation, lung injury, or decreased lung function [40, 41]. Our results demonstrated that GERD in bronchiectasis increased during the 15-year period. Greater lung function loss and increased severity of disease on high-resolution computed tomography were found in patients with GERD-associated bronchiectasis [42]. Therefore, GERD should be considered for patients with bronchiectasis who have no prominent etiology and decreased lung function.

Prominent geographic differences in microbiology have been reported [7]. In our cohort, *P. aeruginosa*, NTM, *H. influenza,* and *K. pneumoniae* were found to be major pathogens. This study was based on the CGRD of real-world clinical practice, and not all patients received sputum culture at the initial diagnosis of bronchiectasis. Our etiology groups exhibited different microbiology spectra. *P. aeruginosa* and *H. influenza* were the major pathogens in the postinfection and COPD groups, whereas NTM was the most common pathogen in post-tuberculosis group. Several groups have reported variable prevalence of NTM infection in Asia (2–4% in southern China and 44.5% in South Korea) [4, 43]. The specific microbiology spectrum of different etiologies has been reported in an Indian population cohort, in which post-infection, post-tuberculosis and idiopathic groups grew more species of cultured bacteria and *P. aeruginosa*, Enterobacteriaceae species and *S. aureus* were the most common pathogens, while NTM was uncommon in the cohort [14]. Chronic infection is associated with increased exacerbation frequency, accelerated rate of lung function decline and increased mortality [12, 13, 44]. In our cohort, some patients developed chronic infection within 1 year of the first bronchiectasis diagnosis, especially in post-infection, COPD and idiopathic groups. Therefore, these patients should be monitored with routine or repeated culture of sputum samples, to obtain possible pathogens of chronic lung infection as well as guidance for future antibiotic therapies and pulmonary rehabilitation.

Patients with bronchiectasis may have multiple comorbidities and exhibit a higher prevalence of hypertension, diabetes mellitus, hyperlipidaemia, osteoporosis and cancer [28, 45]. Cancer incidence was higher among bronchiectasis patients than among other patients (17.0 vs. 12.2 per 1000 person-years) in a large population-based cohort of Taiwan during a 14-year follow-up period [46]. Chronic inflammation and hypoxia in bronchiectasis may be related to cancer development through abnormal proliferation, tissue repair, and metastasis [47, 48]. Our results indicated that the post-infectious and post-tuberculosis subtypes had higher malignancy rates than the other groups.

The strength of this study is that it was a longitudinal cohort study over 15 years, which allowed us to present the trend of age and etiology of bronchiectasis, which were scarcely reported in Asia. Population-based databases in general lack data on laboratory tests, imaging and functional results. The CGRD cohort was representative of patients diagnosed with bronchiectasis in medical centers and regional hospitals across Taiwan, and the benefits of the CGRD were more accurate diagnosis using laboratory data, image studies, and lung function reports.

The limitations of this study are as follows. First, inherent limitations in claims-based analysis include the inability to completely confirm the bronchiectasis diagnosis. The CGRD database is based on real-world clinical practice, and clinicians made the diagnoses of bronchiectasis according to clinical symptoms, history and radiology reports. We identified patients with bronchiectasis by ICD code and a portion of bronchiectasis diagnoses may be based on clinical symptoms and chest radiographs, which has been criticized as not being a reliable diagnostic tool. Second, the tests for serum IgG, IgA, IgM, total IgE, and specific IgE were measured in cases of clinical suspicion of immunodeficiency. Thus, only 1505 patients completed the immunoglobulin tests. Therefore, we may have underestimated the prevalence of immunodeficiency in our cohort. In addition, we identified the etiology mainly by ICD-9/10 codes; thus, some diseases such as allergic bronchopulmonary aspergillosis, Mounier-Kuhn, Williams-Campbell were not listed in ICD-9/10 codes, so we should identify these diseases by searching radiological reports (CT, bronchoscopy), and add an immune screen for these patients in clinical practice in the future. Third, because some parameters were lacking or incomplete from the CGRD, we could not derive the bronchiectasis severity index for disease prediction. Fourth, we were unable to analyze the general prevalence and incidence of bronchiectasis in Taiwan because CGRD didn’t include the entire population. Fourth, the CGRD cohort was from tertiary medical centers and regional hospitals; thus, the patients might have features of more severe bronchiectasis.

## Conclusion

We identified 15,729 patients with a diagnosis claim of bronchiectasis from CGRD over a 15-year period. Postinfection was the main etiology and post-tuberculosis bronchiectasis decreased over the period. The microbiology and comorbidities of bronchiectasis differed in these specific phenotypes. The study provides evidence for ethnic and geographical differences in bronchiectasis and may contribute to the development of more personalized approaches to diagnosis and management in the future.

## Data Availability

The data will not be shared according to the regulations of Chang Gung Memorial Hospital IRB for patient confidentiality.

## Abbreviations

CF: cystic fibrosis
CGMH: Chang Gung Memorial Hospital.
CGRD: Chang Gung Research Database.
COPD: chronic obstructive pulmonary disease.
FEV_1_: forced expiratory volume in one second.
GERD: gastroesophageal reflux.
HRCT: high resolution computed tomography.
ICD: International Classification of Diseases.
NHIRD: national health insurance database.
NTM: Nontuberculous mycobacteria.

## References

[1] Quint JK, Millett ER, Joshi M, Navaratnam V, Thomas SL, Hurst JR (2016). Changes in the incidence, prevalence and mortality of bronchiectasis in the UK from 2004 to 2013: a population-based cohort study. Eur Respir J.

[2] Henkle E, Chan B, Curtis JR, Aksamit TR, Daley CL, Winthrop KL (2018). Characteristics and health-care utilization history of patients with bronchiectasis in US medicare enrollees with prescription drug plans, 2006 to 2014. Chest..

[3] Aksamit TR, O'Donnell AE, Barker A, Olivier KN, Winthrop KL, Daniels MLA (2017). Adult patients with bronchiectasis: a first look at the US bronchiectasis research registry. Chest.

[4] Guan WJ, Gao YH, Xu G, Lin ZY, Tang Y, Li HM (2015). Sputum bacteriology in steady-state bronchiectasis in Guangzhou. China Int J Tuberc Lung Dis.

[5] Buscot M, Pottier H, Marquette CH, Leroy S (2016). Phenotyping adults with non-cystic fibrosis bronchiectasis: a 10-year cohort study in a French regional university hospital center. Respiration..

[6] Olveira C, Padilla A, Martinez-Garcia MA, de la Rosa D, Giron RM, Vendrell M (2017). Etiology of bronchiectasis in a cohort of 2047 patients. An analysis of the Spanish historical bronchiectasis registry. Arch Bronconeumol.

[7] Chandrasekaran R, Mac Aogain M, Chalmers JD, Elborn SJ, Chotirmall SH (2018). Geographic variation in the aetiology, epidemiology and microbiology of bronchiectasis. BMC Pulm Med.

[8] Palwatwichai A, Chaoprasong C, Vattanathum A, Wongsa A, Jatakanon A (2002). Clinical, laboratory findings and microbiologic characterization of bronchiectasis in Thai patients. Respirology..

[9] Steinfort DP, Brady S, Weisinger HS, Einsiedel L (2008). Bronchiectasis in Central Australia: a young face to an old disease. Respir Med.

[10] Guan WJ, Gao YH, Xu G, Lin ZY, Tang Y, Li HM (2015). Aetiology of bronchiectasis in Guangzhou, southern China. Respirology..

[11] Qi Q, Wang W, Li T, Zhang Y, Li Y (2015). Aetiology and clinical characteristics of patients with bronchiectasis in a Chinese Han population: a prospective study. Respirology..

[12] Chalmers JD, Aliberti S, Filonenko A, Shteinberg M, Goeminne PC, Hill AT (2018). Characterization of the "frequent exacerbator phenotype" in bronchiectasis. Am J Respir Crit Care Med.

[13] Hsieh MH, Lin CY, Wang CY, Fang YF, Lo YL, Lin SM (2018). Impact of concomitant nontuberculous mycobacteria and Pseudomonas aeruginosa isolates in non-cystic fibrosis bronchiectasis. Infect Drug Resist.

[14] Dhar R, Singh S, Talwar D, Mohan M, Tripathi SK, Swarnakar R (2019). Bronchiectasis in India: results from the European multicentre bronchiectasis audit and research collaboration (EMBARC) and respiratory research network of India registry. Lancet Glob Health.

[15] Tsai MS, Lin MH, Lee CP, Yang YH, Chen WC, Chang GH (2017). Chang gung research database: a multi-institutional database consisting of original medical records. Biom J.

[16] Shao SC, Chan YY, Kao Yang YH, Lin SJ, Hung MJ, Chien RN (2019). The Chang gung research database-a multi-institutional electronic medical records database for real-world epidemiological studies in Taiwan. Pharmacoepidemiol Drug Saf.

[17] Nicotra MB, Rivera M, Dale AM, Shepherd R, Carter R (1995). Clinical, pathophysiologic, and microbiologic characterization of bronchiectasis in an aging cohort. Chest..

[18] Naidich DP, McCauley DI, Khouri NF, Stitik FP, Siegelman SS (1982). Computed tomography of bronchiectasis. J Comput Assist Tomogr.

[19] Martinez-Garcia MA, de Gracia J, Vendrell Relat M, Giron RM, Maiz Carro L, de la Rosa CD (2014). Multidimensional approach to non-cystic fibrosis bronchiectasis: the FACED score. Eur Respir J.

[20] Celli BR, MacNee W, Force AET (2004). Standards for the diagnosis and treatment of patients with COPD: a summary of the ATS/ERS position paper. Eur Respir J.

[21] Bateman ED, Hurd SS, Barnes PJ, Bousquet J, Drazen JM, FitzGerald JM (2008). Global strategy for asthma management and prevention: GINA executive summary. Eur Respir J.

[22] Deyo RA, Cherkin DC, Ciol MA (1992). Adapting a clinical comorbidity index for use with ICD-9-CM administrative databases. J Clin Epidemiol.

[23] Quan H, Sundararajan V, Halfon P, Fong A, Burnand B, Luthi JC (2005). Coding algorithms for defining comorbidities in ICD-9-CM and ICD-10 administrative data. Med Care.

[24] Finch S, McDonnell MJ, Abo-Leyah H, Aliberti S, Chalmers JD (2015). A comprehensive analysis of the impact of *Pseudomonas aeruginosa* colonization on prognosis in adult bronchiectasis. Ann Am Thorac Soc.

[25] Miller MR, Hankinson J, Brusasco V, Burgos F, Casaburi R, Coates A (2005). Standardisation of spirometry. Eur Respir J.

[26] Bellelli G, Chalmers JD, Sotgiu G, Dore S, McDonnell MJ, Goeminne PC (2016). Characterization of bronchiectasis in the elderly. Respir Med.

[27] Chalmers JD, Goeminne P, Aliberti S, McDonnell MJ, Lonni S, Davidson J (2014). The bronchiectasis severity index. An international derivation and validation study. Am J Respir Crit Care Med.

[28] McDonnell MJ, Aliberti S, Goeminne PC, Restrepo MI, Finch S, Pesci A (2016). Comorbidities and the risk of mortality in patients with bronchiectasis: an international multicentre cohort study. Lancet Respir Med.

[29] Goeminne PC, Hernandez F, Diel R, Filonenko A, Hughes R, Juelich F (2019). The economic burden of bronchiectasis - known and unknown: a systematic review. BMC Pulm Med.

[30] Fu S, Huang N, Chou YJ (2014). Trends in the prevalence of multiple chronic conditions in Taiwan from 2000 to 2010: a population-based study. Prev Chronic Dis.

[31] Lin MH, Chou MY, Liang CK, Peng LN, Chen LK (2010). Population aging and its impacts: strategies of the health-care system in Taipei. Ageing Res Rev.

[32] Byrne AL, Marais BJ, Mitnick CD, Lecca L, Marks GB (2015). Tuberculosis and chronic respiratory disease: a systematic review. Int J Infect Dis.

[33] Taiwan Centers for Diseae Control. Incidence of tuberculosis in Taiwan from 2005–2017. https://daily.cdc.gov.tw/stoptb/CareMagChart.aspx. Accessed 22 July 2019.

[34] Ip MS, So SY, Lam WK, Yam L, Liong E (1992). High prevalence of asthma in patients with bronchiectasis in Hong Kong. Eur Respir J.

[35] Martinez-Garcia MA, Soler-Cataluna JJ, Donat Sanz Y, Catalan Serra P, Agramunt Lerma M, Ballestin Vicente J (2011). Factors associated with bronchiectasis in patients with COPD. Chest..

[36] Du Q, Jin J, Liu X, Sun Y (2016). Bronchiectasis as a comorbidity of chronic obstructive pulmonary disease: a systematic review and meta-analysis. PLoS One.

[37] Patel IS, Vlahos I, Wilkinson TM, Lloyd-Owen SJ, Donaldson GC, Wilks M (2004). Bronchiectasis, exacerbation indices, and inflammation in chronic obstructive pulmonary disease. Am J Respir Crit Care Med.

[38] Martinez-Garcia MA, de la Rosa CD, Soler-Cataluna JJ, Donat-Sanz Y, Serra PC, Lerma MA (2013). Prognostic value of bronchiectasis in patients with moderate-to-severe chronic obstructive pulmonary disease. Am J Respir Crit Care Med.

[39] Jairam PM, van der Graaf Y, Lammers JW, Mali WP, de Jong PA (2015). Group PS. Incidental findings on chest CT imaging are associated with increased COPD exacerbations and mortality. Thorax..

[40] Sweet MP, Patti MG, Hoopes C, Hays SR, Golden JA (2009). Gastro-oesophageal reflux and aspiration in patients with advanced lung disease. Thorax..

[41] Blondeau K, Dupont LJ, Mertens V, Verleden G, Malfroot A, Vandenplas Y (2008). Gastro-oesophageal reflux and aspiration of gastric contents in adult patients with cystic fibrosis. Gut..

[42] McDonnell MJ, Ahmed M, Das J, Ward C, Mokoka M, Breen DP (2015). Hiatal hernias are correlated with increased severity of non-cystic fibrosis bronchiectasis. Respirology..

[43] Park J, Kim S, Lee YJ, Park JS, Cho YJ, Yoon HI (2016). Factors associated with radiologic progression of non-cystic fibrosis bronchiectasis during long-term follow-up. Respirology..

[44] Loebinger MR, Wells AU, Hansell DM, Chinyanganya N, Devaraj A, Meister M (2009). Mortality in bronchiectasis: a long-term study assessing the factors influencing survival. Eur Respir J.

[45] Huang HY, Sheng TF, Lin CW, Wang TW, Lo CY, Chung FT (2019). Oxygen desaturation during the 6-min walk test as a risk for osteoporosis in non-cystic fibrosis bronchiectasis. BMC Pulm Med.

[46] Chung WS, Lin CL, Lin CL, Kao CH (2015). Bronchiectasis and the risk of cancer: a nationwide retrospective cohort study. Int J Clin Pract.

[47] Muz B, de la Puente P, Azab F, Azab AK (2015). The role of hypoxia in cancer progression, angiogenesis, metastasis, and resistance to therapy. Hypoxia (Auckl).

[48] Tong WW, Tong GH, Liu Y (2018). Cancer stem cells and hypoxia-inducible factors (review). Int J Oncol.

